# Supplementary figures and images for: RNG2 tethers the conoid to the apical polar ring in Toxoplasma gondii to enable parasite motility and invasion
Source: PLoS Biol. 2025 Nov 24;23(11):e3003506. doi: 10.1371/journal.pbio.3003506 (PMC12671742; doi:10.1371/journal.pbio.3003506)

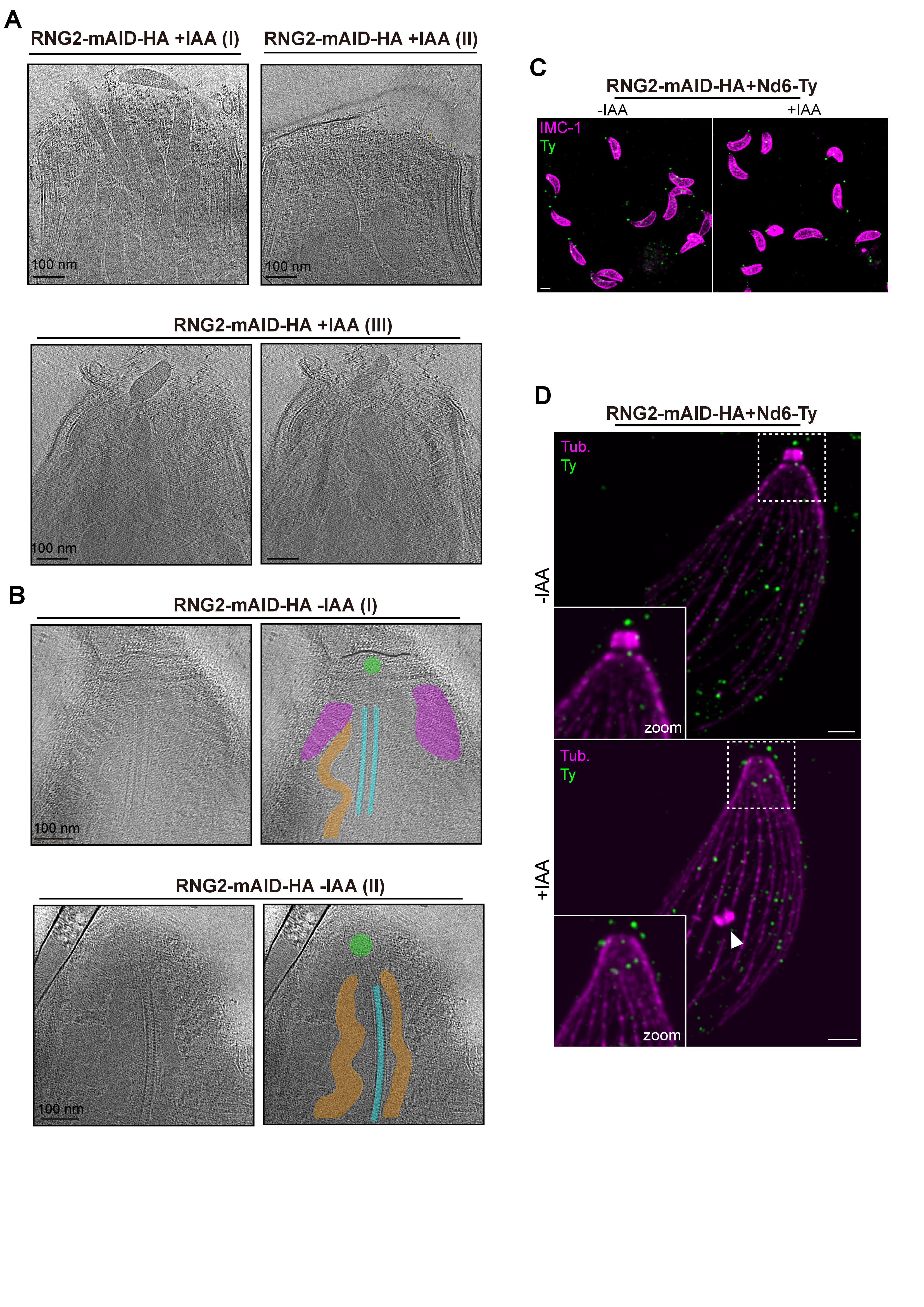

Supplement: S5 Fig — (A) Tomogram slices presented in Fig 4 without any overlay (B) Tomogram slices with and without overlay of untreated RNG2-mAID showing the correct positioning of the elements of the apical complex. (C) IFA images showing the localization of the Nd6 protein in presence or absence of RNG2. (D) U-ExM images showing the localization of the Nd6 protein in with the conoid attached or detached from the apical pole of the parasite. (TIFF) [file pbio.3003506.s005.tiff]

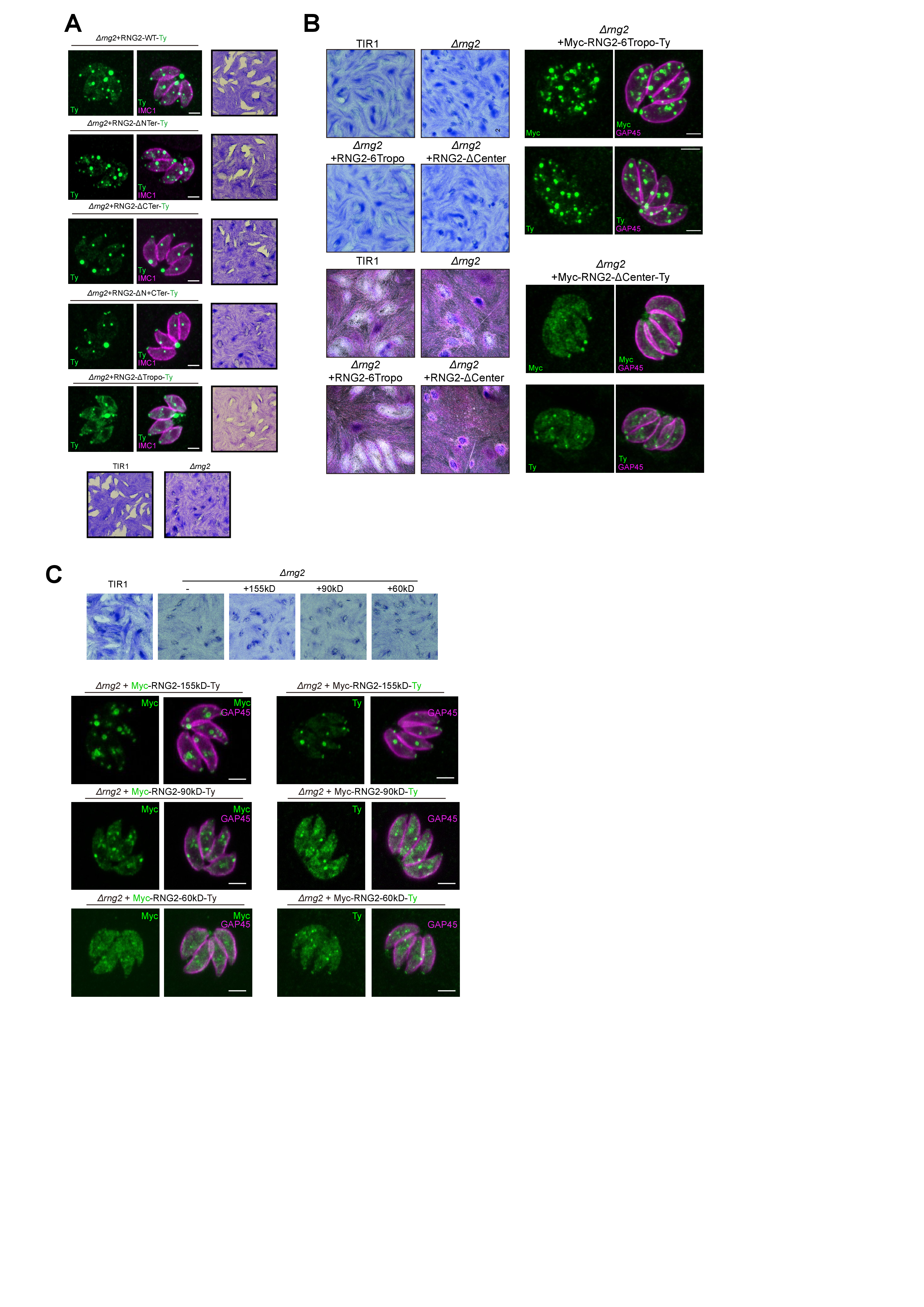

Supplement: S6 Fig — (A) IFA localization and plaque assay assessment of the first round of RNG2 variants. Cell marker: IMC1. Scale bar = 2 μm. (B) IFA localization and plaque assay assessment of the second round of the RNG2 variant. Cell marker: GAP45. Scale bar = 2 μm (C) IFA localization and plaque assay assessment of the processed forms observed in insect cells and expressed in the UPRT locus. Cell marker: GAP45. Scale bar = 2 μm. (TIFF) [file pbio.3003506.s006.tiff]
