# Supplementary material for: RNG2 tethers the conoid to the apical polar ring in Toxoplasma gondii to enable parasite motility and invasion
Source: PLoS Biol. 2025 Nov 24;23(11):e3003506. doi: 10.1371/journal.pbio.3003506 (PMC12671742; doi:10.1371/journal.pbio.3003506)
Supplement: S1 Raw images — (PDF) [file pbio.3003506.s009.pdf]

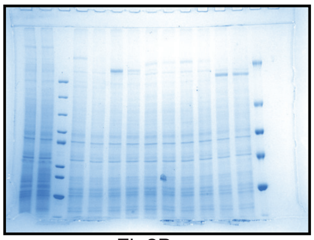
Fig2B

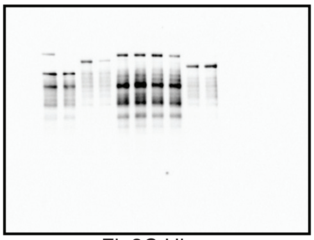
Fig2C His

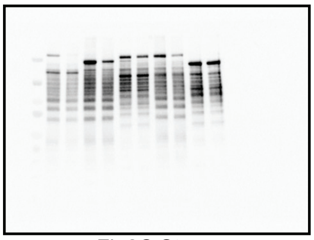
Fig2C Strep

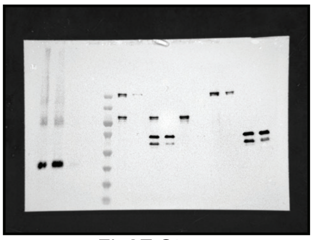
Fig2E Strep

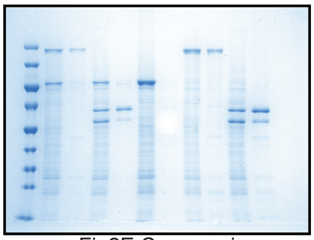
Fig2E Coomassie

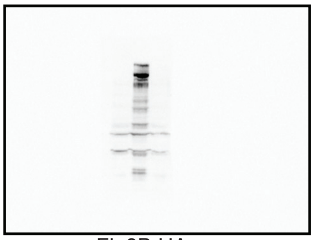
Fig3B HA

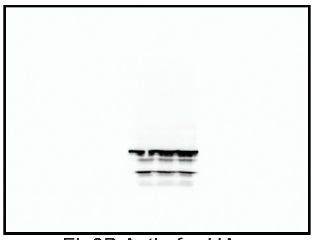
Fig3B Actin for HA

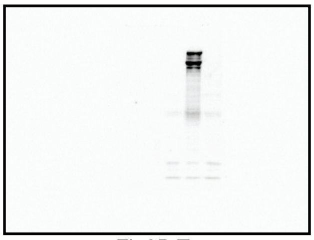
Fig3B Ty

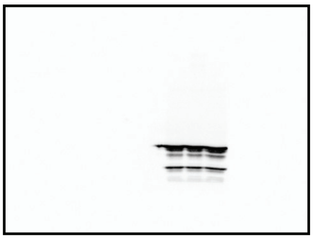
Fig3B Actin for Ty

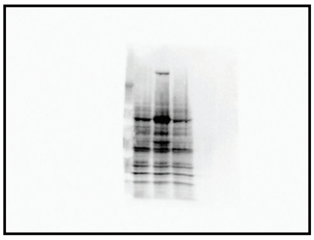
Fig3B Myc

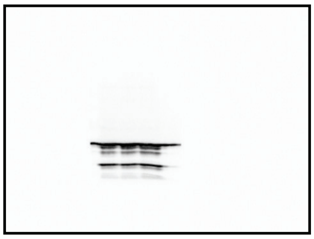
Fig3B Actin for Myc

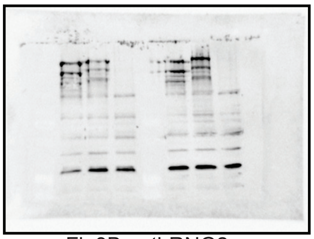
Fig3B anti-RNG2

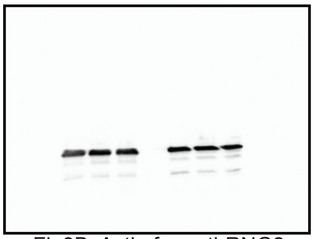
Fig3B Actin for anti-RNG2

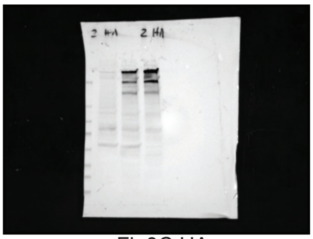
Fig3C HA

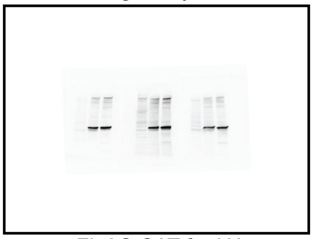
Fig3C CAT for HA

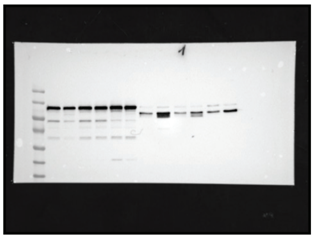
Fig5C MIC2

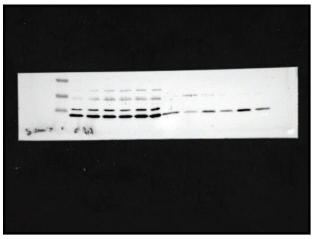
Fig5C GRA1

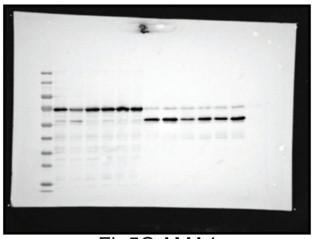
Fig5C AMA1

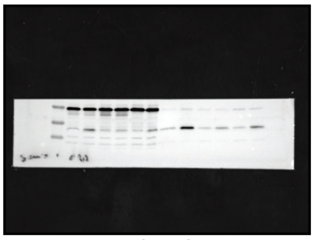
Fig5C MIC6

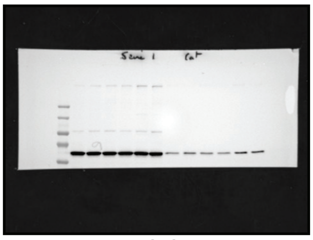
Fig5C CAT

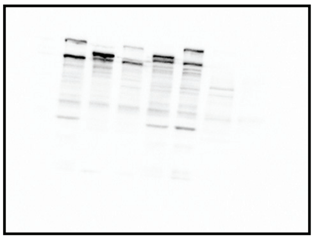
Fig6B Ty

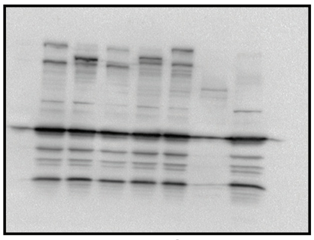
Fig6B CAT

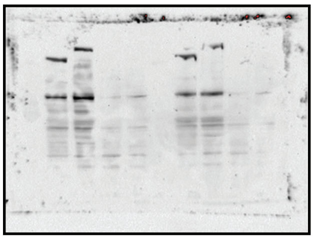
Fig6I Myc

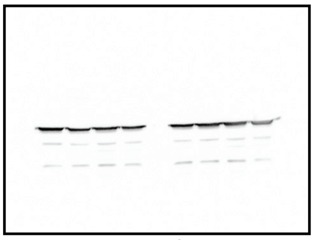
Fig6I Actin for Myc

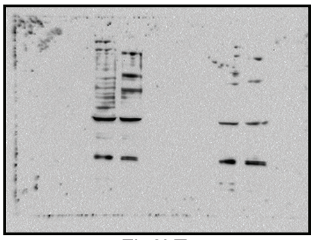
Fig6I Ty

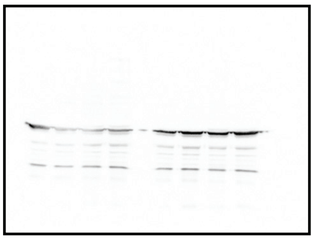
Fig6I Actin for Ty

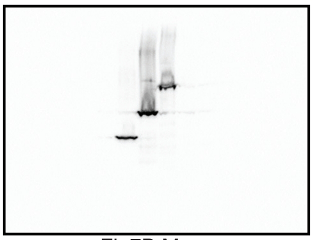
Fig7B Myc

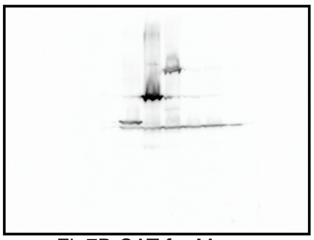
Fig7B CAT for Myc

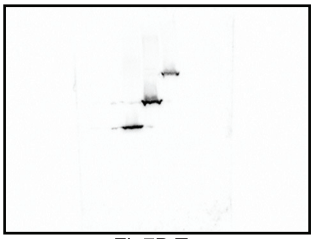
Fig7B Ty

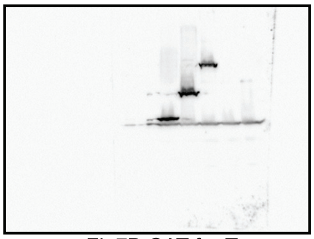
Fig7B CAT for Ty

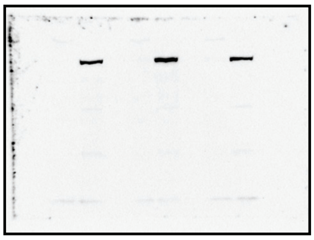
Fig7J WT Ty

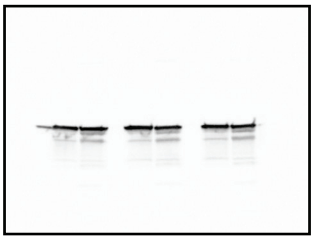
Fig7J Actin for WT Ty

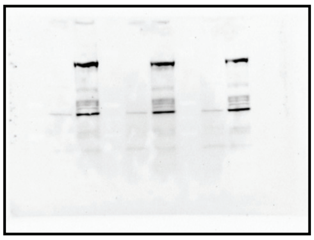
Fig7J WT Myc

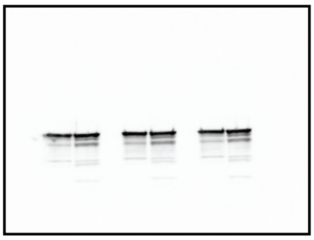
Fig7J Actin for WT Myc

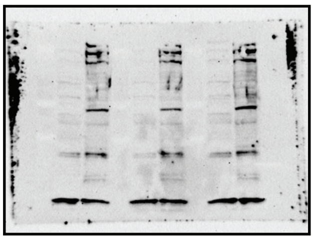
Fig7J MUT Ty

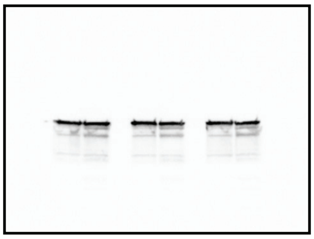
Fig7J Actin for MUT Ty

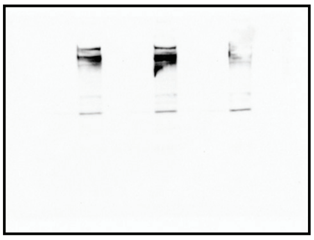
Fig7J MUT Myc

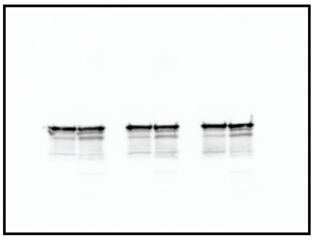
Fig7J Actin for MUT Myc

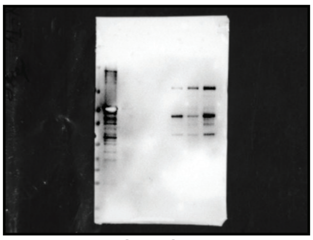
Fig S1B Strep

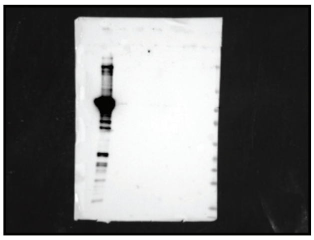
Fig S1B His

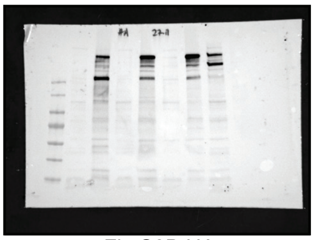
Fig S3B HA

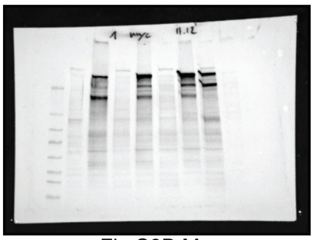
Fig S3B Myc

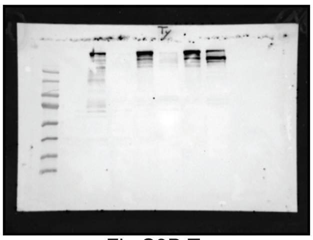
Fig S3B Ty

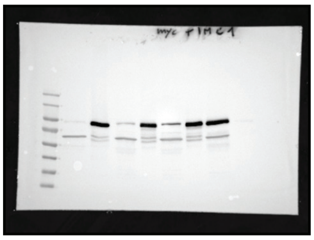
Fig S3B IMC1

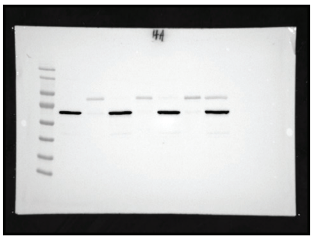
Fig S3B CAT

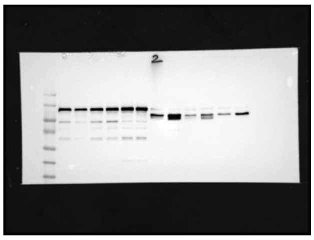
Fig S4B top MIC2

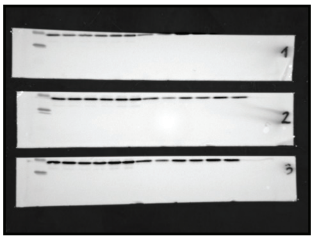
Fig S4B top GRA1

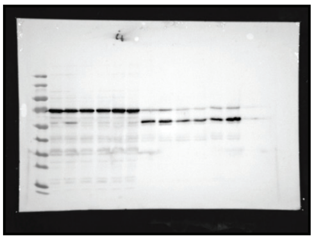
Fig S4B top AMA1

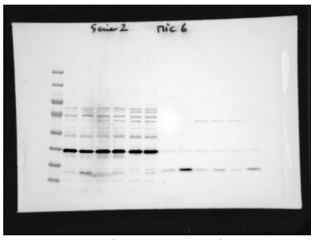
Fig S4B top MIC6

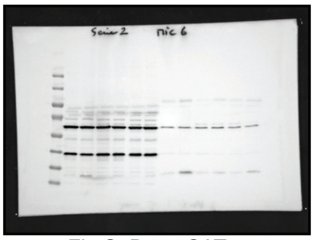
Fig S4B top CAT

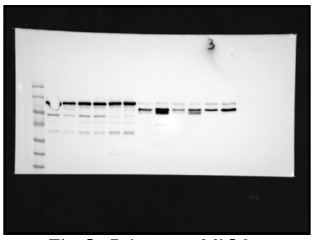
Fig S4B bottom MIC2

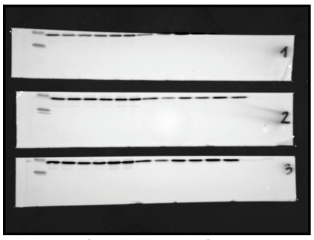
Fig S4B bottom GRA1

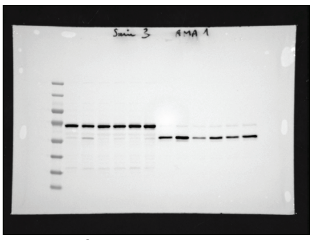
Fig S4B bottom AMA1

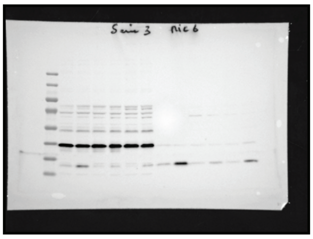
Fig S4B bottom MIC6

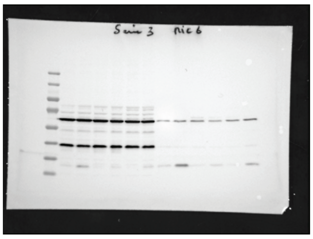
Fig S4B bottom CAT
