# Supplementary material for: RNG2 tethers the conoid to the apical polar ring in Toxoplasma gondii to enable parasite motility and invasion
Source: PLoS Biol. 2025 Nov 24;23(11):e3003506. doi: 10.1371/journal.pbio.3003506 (PMC12671742; doi:10.1371/journal.pbio.3003506)
Supplement: Attachment — Submitted filename: Rebuttal.pdf [file pbio.3003506.s013.pdf]

Dear Dominique,

Please find attached the comments from the 3 reviewers of your manuscript. Please pay special attention to the critique of the in vitro analysis of RNG2 and it reported self-processing, which all reviewers raise. In particular the likelihood of heterologous system proteases playing a role in the cleavage events observed and the inability to see corresponding bands in the lysates of parasites using the N- C- and internal tags. Attention is also warranted on the interpretation of the EM and immunogold labelling as stated by reviewer 1.

We cannot make any decision about publication until we have seen the revised manuscript and your response to the reviewers' comments. Your revised manuscript is also likely to be sent to reviewers for further evaluation.

When you are ready to resubmit, please upload the following:

[1] A letter containing a detailed list of your responses to the review comments and a description of the changes you have made in the manuscript. Please note while forming your response, if your article is accepted, you may have the opportunity to make the peer review history publicly available. The record will include editor decision letters (with reviews) and your responses to reviewer comments. If eligible, we will contact you to opt in or out.

[2] Two versions of the revised manuscript: one with either highlights or tracked changes denoting where the text has been changed; the other a clean version (uploaded as the manuscript file).

Important additional instructions are given below your reviewer comments.

Please prepare and submit your revised manuscript within 60 days. If you anticipate any delay, please let us know the expected resubmission date by replying to this email. Please note that revised manuscripts received after the 60-day due date may require evaluation and peer review similar to newly submitted manuscripts.

Thank you again for your submission. We hope that our editorial process has been constructive so far, and we welcome your feedback at any time. Please don't hesitate to contact us if you have any questions or comments.

Sincerely,

Christopher J. Tonkin
Guest Editor

PLOS Pathogens

Margaret Phillips
Section Editor

PLOS Pathogens

Michael Malim
Editor-in-Chief
PLOS Pathogens

orcid.org/0000-0002-7699-2064

****

Dear Dominique,

Please find attached the comments from the 3 reviewers of your manuscript. Please pay special attention to the critique of the in vitro analysis of RNG2 and it reported self-processing, which all reviewers raise. In particular the likelihood of heterologous system proteases playing a role in the cleavage events observed and the inability to see corresponding bands in the lysates of parasites using the N- C- and internal tags. Attention is also warranted on the interpretation of the EM and immunogold labelling as stated by reviewer 1.

We are grateful for the high-quality reviews provided by the three reviewers. Their time and expertise were instrumental in improving our work.

Part I – Summary

Reviewer #1
Haase et al beautiful extend on the previous observation of the protein RNG2 locating across the gap between the base of the conoid in Toxoplasma and the apical polar ring. They confirm that RNG2 is anchored to both of these structuresand , spectacularly, they show that without wildtype RNG2 the conoid is prone to detach from the cell cytoskeleton during conoid extrusion events. This strongly implicates RNG2 as the main tether holding the mobile element of the cell to the rest of the cell's subpellicular structures. Investigating the behaviour of secretory components, micronemes and rhopties, further implicate the conoid as an instrument that coordinates the positioning of these vesicles, albeit not absolutely essential for all secretion of them. The authors present a range of data types, from biochemical, ultrastructural and experimental cell biological, to interrogate the role and behaviour of RNG2 and its constituent elements. While this commendable body of work provides many supporting observations, there are some elements of the authors' model and conclusion that goes beyond what the data can objectively support, and these warrant some revision. In particular, the evidence for oligomerization, self-processing, and possible functions of these processes in the tethering of the conoid by RNG2 are not convincingly demonstrated with other interpretations of the data possible.

Reviewer #2
In this work, the authors revisited the structural organization and function of RNG2, a protein first discovered and characterized by the Waller Lab. The main new finding in this paper is the role of RNG2 in conoid/APR association. This is a very interesting finding and is well supported by imaging and quantification of conoid detachment when conoid extrusion is induced. The analysis of function complementation of various truncation mutants in the RNG2 knockout background is also solid and informative. However, the interpretation of some other major pieces of data is confusing and sometimes questionable (see below). Additionally, the RNG2 paper from the Waller lab reported that the depletion of RNG2 results in a microneme secretion defect, which is different from the conclusion in this paper. This difference should be explained.

Reviewer #3

This work revisits the location and function of RNG2 in Toxoplasma. The authors confirmed previous conclusions from Katris et al. (2014) study and showed in greater details the correspondence of RNG2 with the 22 SPMTs arising from the APR and provide evidence that RNG2 spans the region between the conoid and the APR. Moreover they provide IEM data suggesting RNG2 association with the Binding Fibers. Conditional depletion of RNG2 induces detachment of the conoid from the APR, a phenotype induced upon actomyosin-dependent extrusion of the conoid, supporting a role of RNG2 in tethering the conoid to APR. They also undertook the biochemical characterization of RNG2 domains, its processing and oligomerization, using recombinant proteins; and performed functional characterization of domains using complementation of RNG2 mutant with truncated tagged version. They conclude that RNG2 is self-processed and oligomerizes in vitro, and propose that the central region of RNG2 is self-processed into small fragments that form filaments to accommodate the flexible distance between the conoid and APR upon extrusion of the conoid.

The presented findings and insights are significant. The quality of the images, in particular, is high. The phenotype of the RNG2 mutant is quite well documented but lacks clarification. The conclusions on the biochemical properties of RNG2 that lead the model of filaments responsible for flexibility of the protein and maintaining the conoid attached to the APR, are the weak part of the paper.

Part II – Major Issues: Key Experiments Required for Acceptance

Reviewer #1

Regarding the conclusion of RNG2 self-processing, and the relevance of this to RNG2 function, from the data described in line 127, can processing by an insect cell protease be eliminated? These results are consistent with degradation yes, but is this a natural process one expects in Toxoplasma? Line 149-152: Are the truncated bands seen in Toxoplasma consistent with the sizes of the putative self-cleaved bands from the insect cell lines. This should be able to test the hypothesis of cleavage seen in the heterologous system being relevant to the parasite, or at least that the sites of cleavage are the same. Size estimates should be given to allow this assessment. Nevertheless, to demonstrate 'self-processing' full-length protein needs to be isolated first, and then subsequent processing observed in physiologically relevant conditions without other cell extracts present.
The notion of self-processing was inappropriately conveyed in the original manuscript. To address the relevance and correspondence of the observed proteolytic cleavages between the in vitro system and the parasite, we have now included a schematic map comparing the major products identified by Western blot in both the in vitro system (insect cells) and *Toxoplasma gondii*. This comparison allows us to estimate the cleavage sites along the protein sequence. Although the sizes of the cleaved products vary depending on the length of the construct expressed (e.g., WT vs. ΔN+Cter), the cleavage sites appear to be conserved between the in vitro system and the parasite. This observation suggests that cleavage likely occurs at regions of increased accessibility or structural fragility of the protein.

What is the evidence that the RNG2 polyclonal is specific to the processed products rather than simply the internal parts of the protein, as stated in the discussion? The strong small fragment in S3B may indicate a dominant internal degradation product that has lost the two termini so is not detected by the end-tagged forms. It is known that the apical complexes of former 'mother cells' during cytokinesis that are broken down after their function. So degraded forms of RNG2 taken from parasite extracts could be sampling this population that is no longer relevant to the cell's apex. Taken together, while it is possible that RNG2 either auto-processes itself, or is prone to processing by some other cell factor, the possibility that auto-processing being important to apical function has not be distinguished from other explanations of the data presented.
The rabbit polyclonal anti-RNG2 antibody was generated using three purified recombinant fragments of RNG2 (155 kDa, 90 kDa, and 60 kDa), which together span the central region of the protein. As a result, this antibody recognizes any RNG2 product containing the central domain, rather than being specific to processed fragments. For example, it reliably detects the full-length RNG2 protein (~300 kDa) in Western blot analyses.
To address concerns about potential degradation from "mother cell" remnants, we compared parasite extracts from intracellular and extracellular parasites by Western blot. We observed no difference in RNG2 processing between these conditions, indicating that the cleavage is not a consequence of recycling old apical complexes, but rather reflects the protein's intrinsic biochemical properties. Together, these data suggest that while RNG2 may undergo proteolytic cleavage, the functional relevance of these processed products cannot be attributed auto-processing, and alternative explanations, including general proteolysis of acesssible regions of the protein.

2. The authors also argue that RNG2 forms oligomers, specifically homo-oligomers with processed forms. Line 132-137: the conclusion for oligomerization in the cell, however, is not sufficiently supported by the data. Elution in the void volume could result from all sorts of aggregation processes in the conditions of these in vitro experiments. The pull-downs do not actually show that oligomers were pulled down, only that the handle was pulled down (the pulldowns might have extracted the handle alone from a complex oligomer/aggregate. In truth, the results obtained here are typically the outcomes one hopes not to get from such protein purification procedures, and appropriate controls are needed to demonstrate that they inform of valid results. Non-denaturing PAGE might be useful to demonstrate the presence of oligomers (as opposed to aggregates) as well as homo-oligomerization of the fragments.
Size-exclusion chromatography (SEC) generally provides higher resolution than non-denaturing PAGE for estimating oligomer size. To increase accuracy, we also ran a panel of control proteins to calibrate the SEC system. Regarding the nature of the RNG2 oligomers, these assemblies were soluble, indicating that they do not form insoluble aggregates or concatamers. Therefore, the elution profiles observed reflect true oligomeric species rather than non-specific aggregation.

3. The immuno-EM attempts an extremely challenging process of testing if the fibres previously resolved between the conoid and apical polar ring are composed of RNG2. This is a likely hypothesis, but this is not easy to test robustly with sufficient controls using immuno-gold EM. One, or even a few, select images consistent with this does not constitute such a test. The authors need to acknowledge this in their report. I think it is fine to include these data as they serve as a

model for how difficult such a question is to answer with these methods, but that these fibres are RNG2 remains a good hypothesis only. Sorry, I do feel your pain!
We fully agree with the reviewer that immuno-gold EM (iEM) is an informative but not definitive approach to demonstrate that the fibers between the conoid and the apical polar ring are composed of RNG2. As such, the iEM data should be interpreted as supportive evidence rather than proof. To strengthen the interpretation, we included iEM of the Glideosome Associated Connector (GAC), which is known to localize at the preconoidal rings. This control shows clear labeling at the expected position, demonstrating the reliability of the iEM technique. Taken together, the U-ExM, iU-ExM, and iEM data provide compelling support for RNG2 as a component of the previously identified binding fibers, while acknowledging that its definitive assignment requires further study.

4. The model shown in Fig 6B appears to conclude that all RNG2 forming the tethers are fragmented and as oligomers. In addition to my concern that these conclusions have not been clearly distinguished from artefacts in the experimental data, is there a need for this model? Is there a reason why the intact RNG2 is considered unable to create the necessary tethering? If so, this should be clearly argued. The phenotype with the shorter version of RNG2 might even argue that its intact length is indeed relevant to function. But at the risk of embedding artefacts into the model, is there actually a need for this layer of complexity?
We thank the reviewer for this very valid point. Our initial model assumed that RNG2 might be too short to span the distance between the conoid and APR, and therefore that proteolytic processing and oligomerization could provide the necessary flexibility. However, distance estimations revealed that full-length RNG2 is indeed sufficient to bridge this space.
To rigorously test the relevance of processing and oligomerization, we performed two new experiments:

1. Transient complementation: Expression of the central part of RNG2 (155 kDa) in a parasite line stably expressing ΔCenter (lacking the central region) did not rescue the phenotype, indicating that separate fragments cannot cooperate to form a functional tether.
2. P2A-mediated splitting: Insertion of a P2A skip peptide to split RNG2 into two fragments during translation, and a mutated version to prevent skipping, showed that split RNG2 cannot complement the phenotype, whereas the mutated full-length version behaves like wild type and fully rescues function.

Collectively, these results demonstrate that RNG2 requires its full length,with the N-terminal domain binding the conoid, the C-terminal region binding the APR, and sufficient length to span the intervening space. Thus, proteolytic cleavage and oligomerization observed in vitro and in parasites are not essential for tethering, and full-length RNG2 alone is sufficient for its function.

Importantly, analysis of in vitro oligomerization revealed that RNG2 fragments assemble exclusively into homo-oligomers of identical size. Such assemblies are thermodynamically favored over mixed-length oligomers (Litowski & Hodges, 2001), suggesting that RNG2 fragments may also preferentially self-associate in the parasite. While these assemblies could contribute to fiber formation, the absence of hetero-oligomers likely explains why fragments cannot substitute for the full-length RNG2 protein.

Reviewer #2

RNG2 self-processing
It appears that when expressed as recombinant proteins in Sf9 cells, the N-terminal tag of various RNG2 construct is lost. It's then concluded that RNG2 self-processes. "Self-process" means that an RNG2 molecule acts on and processes other RNG2 molecules. There is no evidence for this. The loss of N-terminal tag could simply be due to proteolytic degradation by the proteases in the Sf9 cells, which is not surprising given the huge size of RNG2.
Thank you for pointing this. The previous reference to "self-processing" was incorrect. We have rephrased the sentence in the manuscript and now refer to proteolytic cleavages/ degradation instead.

- Related to this, I also don't see any evidence that supports the conclusion that "the processing and oligomerization of the central region of RNG2 are critical for tethering the conoid at the apical pole (L298-299)" or that "RNG2 self-processing is necessary for proper tethering". As far as I can see, there are no processing or oligomerization mutants to test these statements.
Thank you for challenging the model that postulated that processed fragments of RNG2 could oligomerize and help to tether the conoid to APR. To test this hypothesis with have performed two new experiments.

- In transient complementation, we transfected a construct expressing the central part of RNG2 (155kDa) in a parasite line stably expressing a construct lacking the central part of RNG2 (Δcenter). This did not rescue the phenotype suggesting that these two fragments do not cooperate to form a stable tether.
- We have inserted in the middle of the RNG2 protein a P2A sequence (skip peptide) allowing the split of the RNG2 protein into two fragments during translation. In addition, we mutated the P2A to abort the skipping of the ribosome. In the condition while RNG2 was split into two fragments the phenotype was not rescued suggesting that the protein needs to be full length to assume its function. The mutated skip peptide behaved like a WT copy and fully complemented the phenotype.

Importantly, analysis of in vitro oligomerization revealed that RNG2 fragments assemble exclusively into homo-oligomers of identical size. Such assemblies are thermodynamically favored over mixed-length oligomers (Litowski & Hodges, 2001), suggesting that RNG2 fragments may also preferentially self-associate in the parasite. While these assemblies could contribute to fiber formation, the absence of hetero-oligomers likely explains why fragments cannot substitute for the full-length RNG2 protein.

- Line 283-284 states that both the RNG-deltaCenter and 6Tropo variants exhibit several processed forms. Line 295-296 states that RNG-deltaCenter is not processed.
These two statements directly contradict each other.
You are absolutely right, both RNG2-ΔCenter and 6Tropo constructs do undergo processing and the previous statement suggesting otherwise was a mistake. We have corrected the text to ensure accuracy.

2. Counting of RNG2 puncta

In the anti-myc image in Figure 3E, I am counting 28 to 30 RNG2 dots, depending on whether some weaker dots are included. It appears that the authors sometimes count several dots as one puncta (e.g. #4, 15, 19, 20) and sometimes ignore a dot (e.g. one close to #18). What is the criteria for the counting?
In the anti-HA image, the #11 puncta is far removed from the circle that includes other dots. Does this parasite have an aberrant APR?
Thank you for pointing this and indeed this needed clarification. Images displayed are maximum projection of confocal imaging of approximately 100 to 150 slices (depending on the image).
Due to the thickness of the RNG2 signal and the fact that parasites are not always perfectly oriented in top view, a single RNG2 "dot" can appear across multiple slices. Upon projection, this can occasionally result in the appearance of a line or two distinct spots, rather than a single dot.
To reinforce our findings, we performed an additional round of iU-ExM and analyzed 13 new top-view parasites. The majority displayed 22 distinct RNG2 dots, with a range of 21–24, consistent with the organization of the apical polar ring.
Regarding the anti-HA image, we agree that one dot appears slightly displaced. This sample comes from an RNG2-mAID parasite without auxin treatment, which behaves like wild type. The displacement may result from subtle distortions during gelation, where protein anchoring to the gel matrix can be uneven in certain regions.

3. Figure 4G. What is the percentage of Rhoptry mislocalization in RNG2-KD parasites with detached conoid? What is that percentage in RNG2-KD parasites with properly attached conoid?
In RNG2 knockdown parasites, conoid detachment occurs only in extracellular parasites, and is triggered by conoid extrusion upon stimulation (e.g., with BIPPO). Following extrusion, ~80% of RNG2-depleted parasites show conoid detachment.
In wild-type parasites, two rhoptry necks are typically docked within the conoid, aligned for secretion during invasion, and connected to the larger rhoptry bundle. In RNG2-depleted parasites with a detached conoid, rhoptry mislocalization is observed in 100% of cases, the rhoptries remain associated with the detached conoid and are consequently displaced into the cytosol. In contrast, in RNG2-depleted parasites with an attached conoid, rhoptry localization appears normal. This supports the conclusion that rhoptry mislocalization is a direct consequence of conoid detachment.

4. Line 77-78, 309-310: What is the evidence that F-actin enters the pelicular space during motility and that the conoid acts as the gatekeeper for the F-actin entry?
There is substantial evidence in the literature, including work from our own group, showing that F-actin enters the pellicular space during parasite motility, and that the conoid plays a gatekeeping role in this process.
We previously demonstrated that conoid extrusion is required to initiate F-actin flux into the pellicular space, thereby enabling motility and invasion (Dos Santos Pacheco et al., *Nat. Microbiol.*, 2022). More recently, we showed that the apical polar ring (APR) also plays a role by sealing the space between conoid SPMTs, ensuring that F-actin enters the pellicular space rather than diffusing into the cytosol (Ren et al., *PNAS*, 2024). These findings are further supported by cryo-ET data showing the arrangement and directionality of actin filaments during gliding motility in *Toxoplasma* and other Apicomplexans (Martinez et al., *Nat. Commun.*, 2023).

Reviewer #3

1. Overall, the characterization of processing events is confusing

-The cleavage sites in the recombinant are relatively clear, but difficult to be interpreted in the parasites. The western blot showing the triple-tagged RNG2 shows likely processing of RNG2 but it is difficult to reconcile this observation with the data of ΔN+Cter and ΔCter purifications. Despite the presence of a putative processed form at around 250 kDa (Fig. 3B), there is no trace of the 155, or 60, or 90 kDa fragments with the anti-ty antibodies that should recognize the central domain predicted to undergo processing. They also used the anti-RNG2 antibody (against the central part) in the western blot in Fig. S3B but among the many bands, the three fragments of 155, 60, 90 kDa are not clearly detectable (see +IAA vs –IAA). This questions the hypothesis of the authors about the self-processing to produce those specific fragments. The demonstration of several cleavages of the central region within parasites needs to be shown to support their final model.
The reference to self processing in the oringal manuscript was an incorrect statement.
The rabbit polyclonal anti-RNG2 antibody was generated by immunizing with three recombinant fragments (155 kDa, 90 kDa, and 60 kDa), all spanning different parts of the central region of the protein. As a result, this antibody recognizes any product containing the central domain of RNG2. For example, it robustly detects the full-length protein (~300 kDa) in parasite lysates. The processed fragments observed in insect cells were derived from C-terminally truncated RNG2 and therefore migrate at lower molecular weights compared to their corresponding processed forms in parasites**,** which retain more of the C-terminal region. This explains why bands of 155, 90, or 60 kDa are not directly visible in parasite lysates—the processed fragments in parasites are larger and may overlap with other bands or appear at higher molecular weights.
To further support our model, we compared western blots from insect cell-expressed constructs and triple-tagged RNG2 expressed in *T. gondii*. By aligning the sizes of the detected fragments across both systems, we could map five main cleavage sites within RNG2 that are conserved between insect cells and parasites (Fig. S3A). While individual bands are not always sharply resolved in parasite lysates due to protein complexity and post-translational modifications, the pattern is consistent with proteolytic cleavages presumably at conserved regions of accessibility.

However, the functional relevance of the processed fragments has been ruled out. RNG2 must be of sufficient length to serve as a tether between the conoid and the APR, and the cleaved products are not able to compensate for the loss of the full-length protein. This model of tethering via oligomerization has been further invalidated by two new experiments, which demonstrated that cleaved fragments cannot complement one another to restore function. See below the last comments in response to point 1 of this reviewer for more details. We thank the reviewer for prompting us to clarify and test this model more rigorously.

- Fig. 5I, the conclusions on processing of RNG2-6Tropo and ΔCenter constructs are difficult to follow. Lines 283-284 : 'Western blot analysis confirmed that both variants were expressed correctly and exhibited several processed forms, particularly in the extended RNG2 protein.' The western blot clearly shows additional bands in Δcenter lane which the authors themselves claimed to be found also in 6tropo. The many additional bands observed in 6tropo could be degradation

products as also suggested by the strong “unspecific” labeling of round structures within the parasites’ cytoplasm in fig S5C, left panel. It seems that there is more cleavages in Δcenter lane than in 6Tropo lane.
The constructs RNG2 ΔCenter and 6Tropo are indeed processed. Whether the processing is more intense in the 6Tropo because they are more dots observable in the cytosol as consequence of the overexpression (all constructs were expressed under tubulin promoter) is possible. 6Tropo construct seems more prone to proteolytic processing. The decription of the results have been corrected.

-Line 296 ‘RNG2-ΔCenter is not processed, …’. This sentence is confusing, because in the ΔCent construct, there is clear several processing forms.
Thanks for pointing out this contradictory statement which was a mistake. The constructs RNG2 ΔCenter and 6Tropo are processed and the sentence is corrected.

-347-349 Discussion: ‘The colocalization of RNG2-ΔCenter at both the conoid and APR, when antibodies targeted either the N- or C-terminal regions, indicated that RNG2's self-processing is necessary for proper tethering.’ The dual labeling of conoid and APR by the RNG2-ΔCenter might be due to an uneven distribution of the protein when the tearing of the conoid occurs. It is not clear to this reviewer how the observed dual location of the mutant helps this conclusion.
We we agree that clarification was needed. The RNG2-ΔCenter construct retains the domains necessary to bind both the conoid (N-terminus) and the APR (C-terminus), but lacks the central region that provides sufficient length to span the distance between these structures.
In our initial submission, we still let open the possiblitly that RNG2 might be required for tethering. However, our new data rule out this model. Instead, the ΔCenter mutant illustrates that while the binding domains are intact, the truncated protein cannot maintain the physical tether**,** resulting in conoid detachment. The observed dual localization reflects the protein's ability to interact with both structures independently, rather than successful tethering**.**

-287-289 ‘These fragments likely correspond to the minimal regions essential for binding to the conoid and APR, respectively’. I do not fully understand on what this conclusion is based.
We agree that this statement was not sufficiently substantiated by the data and have therefore removed it from the manuscript.

-The authors claim that the oligomers predicted by the gel filtration experiment are not heteroligomers based on data in Fig. 2E. However, from the picture we can see the bands corresponding to the other co-mixed fragments in each of the IPs which might be weak because of the poor enrichment of each tagged protein after IP or because only a fraction of the eluted proteins has been used for the coomassie gel.
To more comprehensively assess RNG2 oligomerization observed in vitro, a new experiment has been performed. Insect cells have been co-infected with different pairs of fragments (155+90, 155+60 ect) and Immunoprecipitation has been performed.
In order to be more resolutive on the possible interaction of these fragments the IP results have been loaded both on a Coomassie gel and for Immuno-blotting. Both results show no significant interactions between the different fragments . In conclusion, the oligomers observed in the void volume of size exclusion chromatography are essentially homo-oligomers.

Overall, I don't think that there are any solid data supporting the conclusion of abstract 'The unique biochemical properties of RNG2, including several proteolytic processing events and its ability to form concatenations, enable it to function as a dynamic bridge between these structures.' and in Lines 295-299 : 'These observations establish ….The processing and oligomerization of the central region of RNG2 are critical for tethering the conoid at the apical pole'. In absence of more direct evidence of cleavage and oligomerization in vivo (not only in vitro), this conclusion should remain speculative.

Thank you for challenging the model that postulated that processed fragments of RNG2 could oligomerize and help to tether the conoid to APR. To test this hypothesis with have performed two new experiments.

- In transient complementation, we transfected a construct expressing the central part of RNG2 (155kDa) in a parasite line stably expressing a construct lacking the central part of RNG2 (Δcenter). This did not rescue the phenotype suggesting that these two fragments do not cooperate to form a stable tether.
- We have inserted in the middle of the RNG2 protein a P2A sequence (skip peptide) allowing the split of the RNG2 protein into two fragments during translation. In addition, we mutated the P2A to abort the skipping of the ribosome. In the condition while RNG2 was split into two fragments the phenotype was not rescued suggesting that the protein needs to be full length to assume its function. The mutated skip peptide behaved like a WT copy and fully complemented the phenotype.

Importantly, analysis of in vitro oligomerization revealed that RNG2 fragments assemble exclusively into homo-oligomers of identical size. Such assemblies are thermodynamically favored over mixed-length oligomers (Litowski & Hodges, 2001), suggesting that RNG2 fragments may also preferentially self-associate in the parasite. While these assemblies could contribute to fiber formation, the absence of hetero-oligomers likely explains why fragments cannot substitute for the full-length RNG2 protein.

2. The authors claimed that that microneme secretion is unaffected in RGN2-depleted parasites, while western blot of Fig. 4B, shows a clear reduction of MIC2 in the media in absence of RNG2, which might be even more pronounced if we take into account the amount of loaded material (Gra1 staining). How many times this experiment has been done is not indicated in the legend. Since, these data tend to show a microneme secretion defect comparable to that observed by Katris et al., a quantification of MIC2's release and of another microneme protein are necessary.

We thank the reviewer for raising this point. To clarify the impact of RNG2 depletion on microneme secretion, we have included two additional biological replicate of these experiments in FigS4 . These show a modest reduction in the secretion of multiple microneme proteins (MIC2, AMA1, MIC6). However, this reduction is not sufficient to account for the major phenotypes observed in RNG2-depleted parasites. For example, these parasites are still able to egress from host cells and lyse both the parasitophorous vacuole membrane (PVM) and host cell, processes that depend on microneme secretion. The primary phenotype of RNG2 depletion arises from defects in motility, due to the absence of the conoid and the associated machinery at the apical pole.

3. In the figure model (Fig. 6A), the authors propose that the two apically docked rhoptries followed the detachment of the conoid and are mislocalized in the cytoplasm. While it is clear that most of rhoptries are found in the cytoplasm in RNG2-depleted parasites (Fig. 4G), one can wonder

if the two rhoptries that are docked at the parasite plasma membrane are also detached, because it is surprising to see only 50% defect in rhoptry secretion. Indeed, this is a mild defect compared to the ARO mutant, which has no rhoptry docked at the apex. TEM should be able to solve this question. This would also allow us to see whether the two internal microtubules and PCRs remain associated at the apex or similarly detach.

We thank the reviewer for this insightful comment. The phenotype observed in RNG2-depleted parasites is dependent on conoid extrusion and retraction. In intracellular parasites, the conoid remains properly positioned. However, during egress and subsequent dissemination, parasites undergo multiple rounds of conoid extrusion/retractions. Timing is critical for interpreting the phenotype. Freshly egressed parasites used for the rhoptry secretion assay do not all have their conoid detached at the same time. Parasites with the conoid in place are still able to secrete rhoptry contents and invade a new host cell, which explains the observed ~50% defect in secretion.

Importantly, in all observations where the conoid was examined, no rhoptries remained apically docked**,** as shown by U-ExM. To consolidate these observations, we performed Cryo-ET experiments. In conoid-less parasites, the only structure that remains at the parasite apex is the Apical Vesicle (AV**)**. These results indicate that the mislocalization of rhoptries in RNG2-depleted parasites is a consequence of conoid detachment rather than partial docking.

Part III – Minor Issues: Editorial and Data Presentation Modifications

Reviewer #1

Immuno-fluorescence images: It would help if the single-channel images were in black and white so that the signals can most clearly be seen, and 3D should have all signals shown separately in this way so that any overlap between them (e.g. Ty and HA) can be clearly assessed. I think this really should be the norm for these types of data. Even though the quantification is shown, these are only for single examples and single 2D trajectories so of useful but limited representation. Thank you for this valuable comment. While we have opted to retain the colored images in the main figures for visual clarity and consistency, we have moved the colocalization data for the different epitope tags to the supplementary information, where each channel is now displayed separately to allow more precise assessment of signal overlap and improves the interpretability of the data.

Line 31: Is a cytoskeletal element and organelle? Some might question this, and 'structure' might avoid raising this question.
Thanks for the suggestion. We have opted to refer to a structure

Line 46: Coccidia should not be in italics according to taxonomic nomenclature, only genus and species names are shown this way.
Corrected.

Line 90: Is it accurate to describe a 'dual localization' when it is only that the two termini are spatially separated as is the case for all proteins to a greater or lesser extent. This description might give the impression that that there are two possible locations that any given protein might adopted. This is a valid point. We have rephrased the sentence to remove the term *"dual localization"* and to clarify that the spatial separation observed reflects the distinct positioning of the N- and C-termini, rather than two alternative localizations of the protein.

Line 115-118 is a little unclear following ',moreover'. Does this relate just to the C-truncations?
The sentence has been rephrased to clarify that only the C-terminal truncations could be purified**,** and this was only achieved under high-salt conditions**.**

Line 208: the 'rubber-band' analogy is not clear from the data cited here.
We agree that the "rubber-band" analogy was not clearly supported by the cited data and may have caused confusion. We have removed it from the text to improve clarity.

For the rhoptry discharge assay, it would be useful to show an example of the images that were used to score positive versus negative. How binary were these observations, and what criteria were used to classify positive versus negative?
Representative images have been added to illustrate the criteria used to classify cells as positive or negative for rhoptry discharge. These examples help clarify how the scoring was performed and show that the observations were sufficiently binary to allow reliable classification.

Line 241: the 'deprived apical F-actin polymerization' is an untested hypothesis, and shouldn't be presented as a conclusion, even if it is considered likely.
We agree that the hypothesis regarding deprived apical F-actin polymerization has not been directly tested. However, the fact that key components of the F-actin flux machinery including Formin1 (normally anchored at the PCRs) and MyoH (normally localized at the conoid) are mislocalized to the cytosol strongly suggests that these parasites are indeed deprived of apico-basal F-actin flux. We have revised the text to present this as a plausible hypothesis rather than a definitive conclusion.

Line 292: 'likel' a typo
Corrected.

Line 296: it is not clear how the conclusion of delta-centre being not processed is reached, nor how this is linked to its mislocalization.
This was indeed a contradictory statements made by mistake The constructs of RNG2 ΔCenter is indeed processed and properly targeted to the APR, not misslocalized. The sentence has been corrected.

Line 298: I do not see how the conclusion of processing being required for tethering is supported by the data.
We thank the reviewer for questioning the model suggesting that processed fragments of RNG2 could oligomerize to tether the conoid to the APR. To address this, we performed additional experiments:

- Transient complementation in trans: We expressed the central part of RNG2 (155 kDa) in a parasite line stably expressing a construct lacking the central part (Δcenter). This did not rescue the phenotype, indicating that the separate fragments do not cooperate to form a stable tether.
- P2A-mediated splitting: We inserted a P2A skip peptide within RNG2 to split the protein into two fragments during translation. In parallel, we mutated the P2A to prevent ribosome skipping. When RNG2 was split, the phenotype was not rescued, suggesting that the full-length protein is required for proper function. The mutated P2A behaved like wild-type RNG2 and fully complemented the phenotype.
- Additionally the P2A-WT could not be complemented by the transient co-expression of the 155kDa fragment.

Taken together, these results support the conclusion that processing events and RNG2 fragements of various siyes are unlikely to hetero-oligomerise and that full-length RNG2 is required for conoid tethering.

Line 306: Apicomplexans is not capitalised, only 'Apicomplexa' when stated as the phylum name.
Corrected.

Line 241-2: Perhaps the conoid detachment phenotype of the BIPPO stimulation is more severe than conoid detachment in normal egress and invasion processes. It would have been nice to test for this witht such a strong stimulus.
We agree that BIPPO represents a strong stimulus. However, conoid detachment is closely coupled to conoid extrusion, and a triggering compound is necessary to ensure synchronized extrusion

across the extracellular parasite population. Compounds such as BIPPO, A23187 (a calcium ionophore), or Zaprinast allow us to assess the phenotype in a controlled and timely manner. Without such stimulation, the low frequency and asynchronous nature of spontaneous conoid extrusion during natural egress or invasion would make the phenotype difficult to capture and quantify.

Reviewer #2

Figure 4C: The labels for "extruded" and "retracted" are difficult to distinguish.
The labels for "extruded" and "retracted" have been updated to improve visibility and ensure clearer distinction between the two conditions.

Reviewer #3

The authors should discuss the fact that Katris et al. (2014) previously didn't observe differences between WT and mutant in the number of extruded conoids, which was likely due to the fact they used phase-contrast microscopy rather than using the more advanced UExM technique.
Conoid extrusion was previously assessed by observing parasite protrusion using phase contrast microscopy. However, this method lacked accuracy and led to the incorrect conclusion that MyoH was not involved in conoid extrusion**.** This limitation was addressed using ultrastructure expansion microscopy (U-ExM), as shown in Dos Santos Pacheco *et al.*, *Nat. Microbiol.*, 2022.

-For gel filtartion experiments, classic markers of known MW such as dextran, thyroglobulin etc, are required to demonstrate that the fractioning of the samples was accurate.
The appropriate MW controls have been injected and now displayed in the figure.

-In Fig. 3D, S2C, S3C. Indicate the significance of white arrows in the legend.
This has been changed, only statistical significant difference ($p < 0.05$) is displayed on the figure

-Fig. S1B, what is "control (+)"?
The "Control (+)" refers to a loading control: an unrelated protein carrying a His-tag, included to demonstrate that the absence of signal in the purifications is not due to a problem with the antibody, but rather to the cleavage of the His-tag from the in vitro–expressed fragments.

-In the text S5C appears before S5B.
The order of the figures has been corrected so that Fig. S5B now appears before Fig. S5C.

-Fig. 4F : Statistic are missing on graph. For non-expert of Toxoplasma egress, images are not understandable without explanation in legend or text.
The statistics displayed in figures are only significant statistical data.

-Fig. 4C, Fig. 5F, S3B miss labeling of Y axis.
The Y axes in Fig. 4C, Fig. 5F, and Fig. S3B have now been correctly labeled.

-Line 92, start by 'here we showed'
We have revised Line 92 to begin with "Here we show…" as suggested.

-Line 208, refer to Fig4B, not the Fig. 4A
Corrected.

-In Western Blots, add arrows and molecular weights to indicate processed products
Arrows and molecular weight markers have now been added to the Western blot figures to indicate the full-length and processed RNG2 products.

-Complementation: the IFAs of complemented lines show a lot of strong staining outside the apical end, indicating mis-localisation. This should be mentioned somewhere in the main text of results.
The IFAs of the complemented lines especially with the small fragment of RNG2 show additional staining outside the apical end, consistent with overexpression. This observation has now been included in the Results section of the main text.

-Fig. S5B, we don't see the lysis plaques. Select better images.
Another set of images from the biological triplicates has been selected and is now displayed alongside the previous ones for comparison.

-There is no M&M of the constructs corresponding to the different recombinant proteins. In particular, the limit boundaries of individual fragments of 60, 90, 155 for baculovirus production are missing.
All RNG2 recombinant fragments were expressed in baculovirus-infected insect cells. Detailed expression and purification protocols have been included in the Materials & Methods section.

-182 Taken together, the iU-ExM- and IEM-based evidence for the spread of RNG2 between the APR support the role of RNG2 as a component of the 22 BFs. Add between the APR 'AND THE CONOID'
Corrected.
